# A novel, rapid method to compare the therapeutic windows of oral anticoagulants using the Hill coefficient

**DOI:** 10.1038/srep29387

**Published:** 2016-07-21

**Authors:** Jeremy B. Chang, Kayla M. Quinnies, Ronald Realubit, Charles Karan, Jacob H. Rand, Nicholas P. Tatonetti

**Affiliations:** 1 Department of Biomedical Informatics, Columbia University Medical Center, New York, New York, USA; 2 Columbia Genome Center, Columbia University Medical Center, New York, New York, USA; 3 Department of Pathology and Laboratory Medicine, New York Presbyterian Hospital, Weill Cornell Medical Center, New York, New York, USA; 4 Departments of Systems Biology and Medicine, Columbia University Medical Center, New York, New York, USA

## Abstract

A central challenge in designing and administering effective anticoagulants is achieving the proper therapeutic window and dosage for each patient. The Hill coefficient, *n*_*H*_, which measures the steepness of a dose-response relationship, may be a useful gauge of this therapeutic window. We sought to measure the Hill coefficient of available anticoagulants to gain insight into their therapeutic windows. We used a simple fluorometric *in vitro* assay to determine clotting activity in platelet poor plasma after exposure to various concentrations of anticoagulants. The Hill coefficient for argatroban was the lowest, at 1.7 ± 0.2 (95% confidence interval, CI), and the Hill coefficient for fondaparinux was the highest, at 4.5 ± 1.3 (95% CI). Thus, doubling the dose of fondaparinux from its IC50 would decrease coagulation activity by nearly a half, whereas doubling the dose of argatroban from its IC50 would decrease coagulation activity by merely one quarter. These results show a significant variation among the Hill coefficients, suggesting a similar variation in therapeutic windows among anticoagulants in our assay.

Traditional anticoagulants such as warfarin require specific dosing for individuals that can be difficult to achieve due in part to a narrow therapeutic window and a pharmacological response that varies greatly from patient to patient^1^,^2^. A response that is too high may cause hemorrhage, whereas a response that is too low would not provide sufficient antithrombotic benefit.

Recently, selective inhibitors of factor Xa and IIa have become available, and these may have wider therapeutic windows as well as less variable pharmacokinetics and pharmacodynamics than warfarin^3^. These include direct thrombin inhibitors such as dabigatran and argatroban; direct factor Xa inhibitors such as apixaban and rivaroxaban; and a synthetic pentasaccharide, fondaparinux. However, a careful side-by-side comparison of the therapeutic windows of these classes of drugs has not been performed.

Here, we consider how one aspect of the dose-response relationship, known as the Hill coefficient or *n*_*H*_, may indicate the drug’s therapeutic window. The Hill coefficient is a measure of the steepness of a dose-response curve, with greater *n*_*H*_ implying a steeper response^4^ (Fig. 1). In the case of anticoagulants acting on the coagulation cascade, a high Hill coefficient implies that a small change in the concentration relative to the half-maximal inhibitory concentration (IC50) can cause a large change in the ability to form a clot. In particular, for an anticoagulant with *n*_*H*_ = 3, maintaining activity within ±10% of half-maximal activity requires that the drug concentration vary at most ~15% from the IC50. In comparison, for *n*_*H*_ = 1, maintaining activity within ±10% of half-maximal activity allows for the drug concentration vary ~40% from the IC50 (Fig. 1). Because pharmacokinetic variation can cause the level of a drug in plasma to vary, a drug with a large Hill coefficient would be expected to cause a more variable response from patient to patient, and a drug with a small Hill coefficient would be expected to have a more consistent response and a wider therapeutic window^2^,^5^. Thus, even if two drugs have the same IC50, one drug may have a wider therapeutic window if its dose-response curve has a lower Hill coefficient.

Measuring dose-response curves can be difficult and time-consuming. Traditional *in vitro* methods of monitoring the activity of the clotting cascade, such as measuring the International Normalized Ratio, are not able to assess the activity of the network as a whole. On the other hand, monitoring the clotting cascade *in vivo* is technically challenging. Therefore, we developed a high-throughput fluorometric assay for thrombin activity, based on similar work from other groups^6^,^7^,^8^,^9^. We used this assay to systematically determine the dose-response relationships and Hill coefficients of five selective anticoagulants in platelet poor plasma.

## Results and Discussion

**Figure 2:**
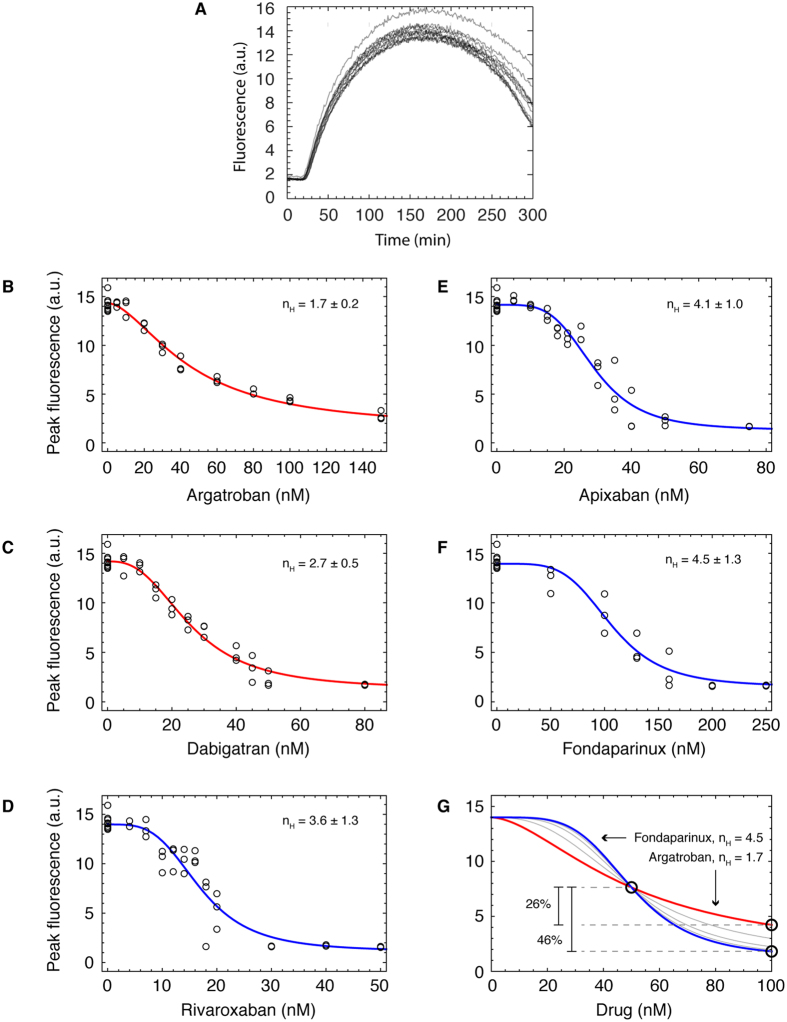
Dose-response curves show variation in Hill coefficient across various anticoagulants. (**A**) A set of 12 control (DMSO-treated) time courses of thrombin activity versus time in platelet poor plasma supplemented with the fluorogenic substrate Z-GGR-AMC and triggered to coagulate using calcium and relipidated tissue factor are shown. Curves are partially opaque; darker portions of the plot indicate the overlap of more than one curve. (**B–F**) The peak fluorescence of each time course at the specified drug concentration are plotted as a function of concentration of (**B**) argatroban, (**C**) dabigatran, (**D**) rivaroxaban, (**E**) apixaban, and (**F**) fondaparinux. Each dose-response curve was fitted to the Hill equation, and these curves are shown in red for thrombin inhibitors (**B,C**) and blue for factor Xa inhibitors (**D–F**). (**G**) The dose-response curves for all drugs is plotted on the same axes and rescaled to have the same IC50, maximum coagulation activity, and minimum coagulation activity in order to compare the differences in the therapeutic windows.

We found that our system was able to adequately capture the dynamics of thrombin generation. We supplemented platelet poor plasma with Z-Gly-Gly-Arg-aminomethylcoumarin (Z-GGR-AMC), which releases fluorescent AMC upon cleavage by thrombin. We initiated coagulation by adding Ca^2+^ and tissue factor, and then we monitored AMC fluorescence over time. A set of 12 time courses of dimethyl sulfoxide (DMSO)-treated control wells is displayed in Fig. 2A. Following the addition of Ca^2+^ and 100 pM lipidated tissue factor, there was a lag phase of about 15 minutes during which the cascade had been triggered, but no detectable thrombin had been generated. The lag phase was followed by an explosive growth in the amount of AMC fluorescence (and therefore thrombin) generated, which continued to rise until its peak at around 170 minutes. After the peak, the fluorescence decayed slowly, presumably due to photobleaching effects. These data show that our assay successfully reproduced the coagulation cascade in an *in vitro*, high throughput assay format.

Subsequently, we determined the dose-response relationships of five different anticoagulants: two thrombin-selective inhibitors (argatroban and dabigatran) and three factor Xa-selective inhibitors (fondaparinux, apixaban, and rivaroxaban). We used the maximum level of fluorescence reached over the entire time course in order to compare thrombin activity at different drug doses, including DMSO-treated controls. In order to determine the Hill coefficient, we fit a Hill curve to the data. These data and the results of the modeling are shown in Fig. 2B–F and summarized in Table 1. Although the plots show only a portion of the dose-response curve for clarity, all drugs span the entire dynamic range from the baseline control level to essentially no AMC fluorescence produced. The best-fit Hill curves are shown in red, and the Hill coefficients ranged from 1.7 ± 0.2 (95% CI) for argatroban to 4.5 ± 1.3 for fondaparinux. Thus, doubling the dose of fondaparinux from its IC50 would lead to a large 46% decrease in coagulation activity, whereas doubling the dose of argatroban from its IC50 would lead to a decrease of 26%, about half the size. This implies that the therapeutic window of argatroban may be significantly greater than that of fondaparinux.

Our method applies a classical enzymatic analysis to develop a novel and rapid method for comparing the therapeutic windows of anticoagulants in plasma. In the context of route of administration, these results are of differing importance for each of these drugs. Some medications are given orally, on a regular basis, while others are administered intravenously. For example, fondaparinux is often administered intravenously^10^, but can sometimes be given orally in low doses^11^. Rivaroxaban is administered orally for initial treatment of venous thromboembolism and secondary prevention after acute coronary syndromes. A major challenge with rivaroxaban and others of similar administrative routes and chemical composition (like dabigitran and apaxiban, also evaluated in this paper) is keeping the maximum plasma concentration (C_max_) below a level with a high bleeding risk, while keeping the minimum plasma concentration (C_trough_) high enough to be effective^12^. Our results provide new insight for predicting these outcomes. We have shown that there is an approximately three-fold variation in the Hill coefficient among the five anticoagulants tested. This implies that certain anticoagulants could have a significantly wider “sweet spot” for delivering a beneficial bleeding risk/antithrombotic benefit ratio.

Our study indicates that some drugs may be more beneficial than others, depending on clinical and patient circumstances. Argatroban and dabigitran have smaller confidence intervals around the Hill coefficient and less severe consequences for increasing the dose. Perhaps medication that meets these criteria should be considered above others, and drugs that have a shorter and more severe decline might be more appropriate for more acute treatment. For example, argatroban has a wide window (Fig. 2B) which has implications for administration, especially since this drug is given intravenously to stroke patients^13^,^14^.

Additional factors can influence choice of anticoagulant. For instance, because dabigitran is eliminated via the kidneys, poor kidney function must be considered for deciding drug and dosage^15^. Furthermore, the experiments in this study were conducted on blood without the presence of additional drugs, whereas patients taking anticoagulants are likely to also take additional medications. When taken concomitantly, the plasma concentration of the anticoagulants may change significantly. Our analysis suggests that anticoagulants with lower Hill coefficients will be more resilient to this type of drug interaction. Study of these drugs in clinical settings are necessary to determine whether this is indeed the case. Indeed, the use of the Hill coefficient as an indicator of therapeutic window should be evaluated in further modeling studies and with experiments *in vitro* and *in vivo*.

## Materials and Methods

### *In vitro* assay for thrombin activity

~100 ml of blood was withdrawn from Dr. Nicholas Tatonetti (N.P.T.) via venipuncture into a standard syringe, and then expelled into tubes with final concentration of 0.011 M sodium citrate and 50 mg/ml corn trypsin inhibitor (SCAT-27-45/50SC, Haematologic Technologies Inc.). Whole blood was immediately processed into platelet poor plasma as follows. Tubes were centrifuged at 2900 × *g* for 20 minutes in an Eppendorf 5810 R table top centrifuge. The supernatant was slowly and carefully removed by pipetting with a trimmed P1000 pipette tip and then transferred into fresh 50 ml screw-top tubes (Corning 430828). These tubes were again centrifuged at 290 × *g*. The supernatant was removed as before, placed into fresh 50 ml tubes, mixed by inversion several times, aliquoted into 15 ml screw-top tubes (Corning 430790), and then frozen by placing into a −80 °C freezer for storage. Before use, aliquots were thawed by immersion in a 37 °C water bath until the tube temperature reached at least room temperature.

Dabigatran (S2196), Rivaroxaban (S3002), and Apixaban (S1593) were purchased from Selleckchem; Fondaprinux sodium (114870-03-0) was purchased from BOC Sciences; and Argatroban monohydrate (A0487-5MG) was purchased from Sigma-Aldrich. All were diluted to 1 mM and 10 mM stock solutions in DMSO.

In order to monitor thrombin activity, 200 μM final concentration of Z-GGR-AMC (Bachem 4002155) was first added to thawed platelet poor plasma from above. 18 μL of this solution was plated into each well of a 384-well plate using a Matrix WellMate 8-channel Microplate Dispenser. Drugs at the desired concentrations and the appropriate amount of DMSO normalization was added to each well using an HP D300 Digital Dispenser. Finally, 1 μL of a solution of 400 mM Ca^2+^ and 100 pM tissue factor (from a lyophilized stock resuspended in 2.6 mM phosphatidylcholine and 2.6 mM phosphatidylserine to 371 nM recombinant tissue factor, RLTF-0350, Haematologic Technologies Inc.) was added using a FlexDrop PLUS Precision Reagent Dispenser to each well to trigger coagulation. After five minutes, the plate was immediately transferred to an Envision 2104-0010 Multilabel Plate Reader to monitor the amount of AMC present at each time (excitation 346 nm and emission 443 nm using a monochromator).

### Data analysis

Data was prepared using custom Python scripts. Curve fitting of dose-response data to the Hill equation was performed in Mathematica (Wolfram).

### Ethics

All methods were carried out in accordance with the protocol, which was reviewed and approved by the Columbia University Medical Center Institutional Review Board (IRB-AAAQ3001). Informed consent was obtained from all subjects.

## Additional Information

**How to cite this article**: Chang, J. B. *et al*. A novel, rapid method to compare the therapeutic windows of oral anticoagulants using the Hill coefficient. *Sci. Rep.* **6**, 29387; doi: 10.1038/srep29387 (2016).

## Figures and Tables

**Figure 1:**
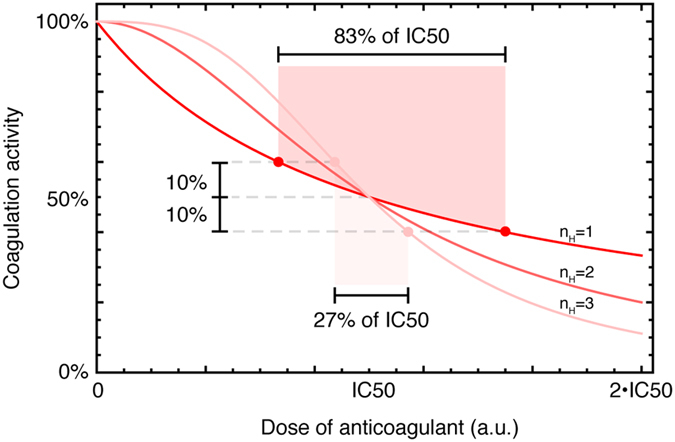
Simulation of anticoagulant dose and coagulation activity in relation to Hill coefficients and therapeutic window. Three simulations with *n*_*H*_ = {1,2,3} of the dose-response relationship of a hypothetical anticoagulant with fixed IC50 and dynamic range. In order to maintain a coagulation activity of 50 ± 10%, the dose of the anticoagulant may vary 83% of the IC50 for *n*_*H*_ = 1, but it may only vary 27% of the IC50 for *n*_*H*_ = 3.

**Table 1:** Parameters resulting from fitting Hill curves to dose-response data.

Drug name	Hill coefficient,*n*_*H*_, 95% CI	IC50(nM)	Percent decreasein activity ifconcentrationdoubles (95% CI)
Apixaban	4.1 ± 1.0	29	44% (40–47%)
Argatroban	1.7 ± 0.2	45	26% (24–29%)
Dabigatran	2.7 ± 0.5	26	37% (32–40%)
Fondaparinux	4.5 ± 1.3	107	46% (40–48%)
Rivaroxaban	3.6 ± 1.3	17	42% (33–47%)
